# Letter to the editor: lansoprazole interferes with fungal respiration and acts synergistically with amphotericin B against multidrug-resistant *Candida auris*

**DOI:** 10.1080/22221751.2024.2356144

**Published:** 2024-05-14

**Authors:** Meng Zhu, Hongyang Wang, Yongsheng Zhang, Fuzhen Pan

**Affiliations:** a School of Basic Medical Sciences, Zhejiang Chinese Medical University, Hangzhou, People’s Republic of China; b Cancer Center, Shenzhen Hospital (Futian) of Guangzhou University of Chinese Medicine, Shenzhen, People’s Republic of China

**Keywords:** Lansoprazole, Antifungal Resistance, Candida auris, Amphotericin B, Proton Pump Inhibitor

## Abstract

The study investigates the potential of lansoprazole, a proton pump inhibitor, to interfere with fungal respiration and enhance the antifungal activity of amphotericin B against multidrug-resistant Candida auris. The authors administered lansoprazole at concentrations significantly higher than typical therapeutic doses, which demonstrated promising results but also raised concerns about potential toxicity. We suggest incorporating a control group, monitoring toxicity indicators, performing pathological examinations, and conducting cellular assays to improve the study's rigor and reliability. We also highlight the need for further research into the mechanisms of lansoprazole's antifungal activity, its long-term effects on amphotericin B resistance, and potential drug-drug interactions with amphotericin B. Addressing these concerns is crucial for the clinical translation of lansoprazole as an adjuvant to amphotericin B.

Dear Authors,

We are writing to express my interest in your recent article titled “lansoprazole interferes with fungal respiration and acts synergistically with amphotericin B against multidrug-resistant *Candida auris* [1].” The study is of great importance, particularly in addressing the challenge of antifungal resistance, which has significant implications for public health. However, after carefully reviewing the article, we have some concerns regarding the potential toxicity of the high dose of lansoprazole used in your study. We would like to share my thoughts and propose some suggestions for further improving the article.

In your study, lansoprazole was administered at concentrations ranging from 16 μM to 300 mg/kg, which is significantly higher than the typical therapeutic dose range. While this approach demonstrated promising results in enhancing the antifungal activity of amphotericin B, it also raises concerns about potential toxicity, which could confound the interpretation of the results.

Based on the FDA Adverse Event Reporting System (FAERS) database, we conducted a disproportionality analysis to identify adverse events that occur more frequently with Lansoprazole compared to other placebos. This involved calculating reporting odds ratios, proportional reporting ratio, Bayesian confidence propagation neural network, and multi-item gamma Poisson shrinker to determine if any adverse events are significantly associated with Lansoprazole. As a result, lansoprazole has been associated with various adverse effects, including kidney disease, excessive stomach acid production, diarrhea, diabetes, hypertension, liver and gallbladder diseases, reproductive system disorders, and immune system disorders (Table 1). The high dose of lansoprazole used in your study may lead to increased toxicity, potentially affecting the health of the animal models and influencing the outcome of the experiments. To address these concerns, we would like to suggest several measures to improve the study’s rigour and enhance the reliability of the results:

1. Incorporating a control group: Consider adding a control group with a lower dose of lansoprazole or a placebo to compare the toxicity and efficacy of different lansoprazole concentrations. This would help to ensure that the observed effects are not solely due to lansoprazole's toxicity.
2. Monitoring toxicity indicators: During the experiment, it would be beneficial to monitor toxicity indicators, such as renal and liver function, reproductive system function, and immune system parameters. This would provide insights into the potential toxic effects of lansoprazole at different doses. Utilize statistical methods to analyze the differences in toxicity between different lansoprazole dose groups. This would enable a quantitative assessment of the relationship between lansoprazole dose and toxicity.
3. Performing pathological examinations: Post-experiment pathological examinations of the animal models’ organs could reveal any pathological changes caused by lansoprazole. This would help to assess the potential organ-specific toxicity of lansoprazole.
4. Cellular assays to evaluate the cytotoxic effects of lansoprazole at various concentrations, which will help in understanding its impact on cellular health and viability.

**Table 1.** Signal detection for lansoprazole – associated adverse events.

SOC	PT	Case reports	ROR (95% CI)	PRR (95% CI)	chisq	BCPNN	MGPS
Renal and urinary disorders	Hypertensive nephropathy	571	593.3 (524.38, 671.27)	590.04 (524.58, 663.67)	148543.99	8.03 (7.88)	261.58 (235.91)
Renal and urinary disorders	Malignant renal hypertension	7	297.91 (115.48, 768.51)	297.89 (116.27, 763.2)	1265.76	7.51 (6.32)	182.43 (82.55)
Renal and urinary disorders	Diabetic end stage renal disease	16	220.32 (121.61, 399.15)	220.29 (122.36, 396.61)	2375.01	7.23 (6.44)	150.11 (91.3)
Renal and urinary disorders	Ischaemic nephropathy	18	200.65 (115.51, 348.55)	200.62 (115.89, 347.31)	2502.65	7.14 (6.39)	140.73 (88.66)
Renal and urinary disorders	Perinephric oedema	4	170.23 (54.2, 534.62)	170.22 (54.61, 530.54)	493.47	6.97 (5.52)	125.1 (48.01)
Renal and urinary disorders	Diabetic nephropathy	538	169.21 (153.31, 186.77)	168.34 (152.63, 185.67)	65826.95	6.96 (6.82)	124.08 (114.24)
Renal and urinary disorders	Renal artery arteriosclerosis	31	163.1 (108.37, 245.47)	163.05 (108.04, 246.08)	3702.94	6.92 (6.36)	121.19 (86.08)
Renal and urinary disorders	End stage renal disease	4149	157.57 (152.07, 163.27)	151.32 (145.5, 157.37)	468366.75	6.84 (6.79)	114.6 (111.24)
Renal and urinary disorders	Chronic kidney disease	14545	140.06 (137.38, 142.8)	120.6 (118.26, 122.99)	1373779.16	6.59 (6.56)	96.1 (94.56)
Renal and urinary disorders	Nephrosclerosis	232	129.11 (111.64, 149.33)	128.83 (112.31, 147.78)	23075.73	6.66 (6.46)	101.24 (89.64)
Renal and urinary disorders	Obstructive nephropathy	18	106.68 (63.93, 177.99)	106.66 (64.07, 177.55)	1534.4	6.44 0(5.73)	87.05 (56.72)
Renal and urinary disorders	Kidney small	47	102.86 (75, 141.05)	102.81 (75.13, 140.68)	3885.21	6.4 (5.96)	84.48 (64.86)
Renal and urinary disorders	Renal hypertension	49	94.44 (69.47, 128.37)	94.39 (68.98, 129.16)	3767.97	6.3 (5.87)	78.72 (60.89)
Renal and urinary disorders	Hypocitraturia	7	74.48 (33.55, 165.35)	74.47 (33.34, 166.33)	437.75	6.01 (4.93)	64.39 (33.03)
Gastrointestinal disorders	Enterochromaffin cell hyperplasia	10	520.17 (211.36, 1280.15)	520.12 (211.13, 1281.34)	2454.27	7.95 (6.89)	246.9 (116.21)
Gastrointestinal disorders	intrinsic factor deficiency	5	260.07 (87.16, 776.05)	260.06 (86.77, 779.39)	829.49	7.39 (6.02)	167.54 (67.12)
Gastrointestinal disorders	Hypergastrinaemia	26	176.43 (112.38, 277)	176.39 (112.38, 276.86)	3293.31	7 (6.39)	128.39 (88.03)
Gastrointestinal disorders	Hyperchlorhydria	890	89.46 (83.25 96.13)	88.7 (82.01 95.93)	64885.84	6.22 (6.12)	74.73 (70.36)
Gastrointestinal disorders	Gastric dysplasia	6	85.12 (35.66 203.14)	85.11 (35.93 201.61)	422.01	6.17 (5.01)	72.17 (34.85)
Gastrointestinal disorders	Rebound acid hypersecretion	445	81.46 (73.65 90.1)	81.12 (73.55 89.47)	30013.76	6.11 (5.97)	69.28 (63.68)
Neoplasms benign malignant and unspecified (incl cysts and polyps)	Renal haemangioma	399	471.09 (409.96 541.33)	469.29 (409.13 538.3)	93108.37	7.88 (7.7)	234.85 (209.07)
Neoplasms benign malignant and unspecified (incl cysts and polyps)	Gastric neuroendocrine carcinoma	3	127.67 (35.62 457.64)	127.67 (35.71 456.44)	296.23	6.65 (5.06)	100.52 (34.54)
Neoplasms benign malignant and unspecified (incl cysts and polyps)	Renal hamartoma	8	78.02 (36.91 164.93)	78.02 (37.05 164.31)	521.36	6.07 (5.05)	67.02 (35.82)
Skin and subcutaneous tissue disorders	Erythema dyschromicum perstans	8	144.04 (65.21 318.17)	144.03 (65.76 315.46)	868.95	6.79 (5.73)	110.38 (56.87)
Skin and subcutaneous tissue disorders	Papulopustular rosacea	5	83.59 (32.28 216.5)	83.59 (31.99 218.4)	346.19	6.15 (4.9)	71.08 (32.06)
Investigations	Urine magnesium decreased	7	655.4 (208.01 2065.06)	655.35 (206.18 2083.01)	1905.62	8.1 (6.83)	273.65 (104.75)
Investigations	Urine phosphorus decreased	6	80.25 (33.75 190.8)	80.25 (33.88 190.1)	400.84	6.1 (4.95)	68.65 (33.26)
Musculoskeletal and connective tissue disorders	Chronic kidney disease-mineral and bone disorder	146	78.67 (66.01 93.75)	78.56 (65.86 93.72)	9572.83	6.07 (5.83)	67.41 (58.21)
Metabolism and nutrition disorders	Hypozincaemia	6	87.78 (36.7 209.93)	87.77 (37.05 207.91)	433.42	6.21 (5.05)	74.07 (35.71)
Endocrine disorders	Hypoparathyroidism secondary	14	81.93 (46.43 144.56)	81.92 (46.4 144.62)	952.37	6.13 (5.34)	69.87 (43.44)

Abbreviations: SOC system organ classes; PT preferred term; ROR reporting odds ratio; PRR proportional reporting ratio; BCPNN Bayesian confidence propagation neural network MGPS multi-item gamma Poisson shrinker; and CI confidence interval.

As to other aspects, firstly, the study does not provide sufficient evidence to confirm that the inhibition of mitochondrial complex III by lansoprazole is the sole or primary mechanism of its antifungal activity. It would be beneficial to investigate and rule out other potential mechanisms that may contribute to its efficacy [2]. Additionally, the study lacks investigation into the long-term effects of lansoprazole on amphotericin B resistance. Assessing the durability of the synergistic effect over an extended period would provide valuable insights into its clinical potential [3]. Furthermore, the exact mechanisms by which lansoprazole enhances the activity of amphotericin B are not fully explained. Exploring and elucidating these mechanisms would strengthen the scientific foundation of the study [4]. Moreover, the potential drug–drug interactions between lansoprazole and amphotericin B have not been studied. Investigating possible interactions is essential for understanding the safety and efficacy of their combined use. The pharmacokinetic properties of lansoprazole, including its metabolism in the human body, have not been thoroughly examined. Further investigations are required to develop a detailed treatment plan and assess the long-term antifungal efficacy of the combination therapy.

In conclusion, while your study has made significant contributions to the field of antifungal resistance, it is essential to address the potential toxicity of the high dose of lansoprazole used. By incorporating the suggested measures, you could enhance the rigour of your study and provide more reliable data to support the clinical translation of lansoprazole as an adjuvant to amphotericin B.
